# Comparison of 68Ga-DOTANOC and 18F-FDG PET-CT Scans in the Evaluation of Primary Tumors and Lymph Node Metastasis in Patients With Rectal Neuroendocrine Tumors

**DOI:** 10.3389/fendo.2021.727327

**Published:** 2021-09-01

**Authors:** Zhihao Zhou, Zhixiong Wang, Bing Zhang, Yanzhang Wu, Guanghua Li, Zhao Wang

**Affiliations:** ^1^Department of Gastrointestinal Surgery, First Affiliated Hospital of Sun Yat-sen University, Guangzhou, Guangdong, China; ^2^Department of Nuclear Medicine, First Affiliated Hospital of Sun Yat-sen University, Guangzhou, Guangdong, China

**Keywords:** rectal neuroendocrine tumors, lymph node metastasis, 68Ga-DOTANOC PET, 18F-FDG PET, PET-CT

## Abstract

**Background:**

Lymph node metastasis of rectal neuroendocrine tumors (RNETs) predicts poor prognosis. However, the assessment of lymph node metastasis remains a challenge. It has been reported that 68Ga-DOTANOC and 18F-FDG PET-CT scans could be employed in the work-up of rectal neuroendocrine tumors (RNETs). This study aimed to assess both tracers’ ability to identify primary tumors and lymph node (LN) metastasis in RNETs.

**Methods:**

A total of 537 patients with RNETs were enrolled from January 2014 to January 2021. Both 68Ga-DOTANOC and 18F-FDG PET-CT scans were used to evaluate primary tumors and LN group metastasis. PET images were evaluated through visual and semiquantitative assessment. Receiver Operating Characteristics (ROC) curve analysis was used to investigate the performance of SUVmax of 68Ga-DOTANOC and 18F-FDG PET in predicting LN group metastasis.

**Results:**

Fifty-two patients with preoperative 68Ga-DOTANOC with 18F-FDG PET-CT scans underwent endoscopic biopsy or dissection of the primary tumor, while 11 patients underwent rectal surgery together with regional LN dissection. For primary tumors, 68Ga-DOTANOC had a sensitivity of 89.58% and a positive predictive value (PPV) of 95.56% through visual assessment, while 18F-FDG PET-CT showed 77.08% sensitivity and 97.37% PPV. For the prediction of LN group metastasis, 68Ga-DOTANOC PET-CT had 77.78% sensitivity and 91.67% specificity, while 18F-FDG PET-CT had 38.89% sensitivity and 100% specificity according to visual assessment. The area under the ROC curves (AUC) for 68Ga-DOTANOC PET/CT was 0.852 (95%CI:0.723-0.981) with an optimal SUVmax cut-off value of 2.25, while the AUC for 18F-FDG PET were 0.664 (95%CI:0.415-0.799) with an optimal SUVmax cut-off value of 1.05.

**Conclusions:**

This study showed that 68Ga-DOTANOC PET-CT was a promising tool for detecting LN metastasis in RNETs with high sensitivity and specificity in visual assessment and semiquantitative assessment, which was better than 18F-FDG PET-CT.

## Introduction

Neuroendocrine tumors (NETs) are considered rare tumors and constitute only 0.5% of all malignant conditions (1). NETs can arise in different organs, including the gastrointestinal tract, pancreas, lungs, gallbladder, thymus, thyroid gland, testes, ovary, and skin (2). Rectal NETs (RNETs) only account for 1% to 2% of rectal tumors (3). In 2010, the World Health Organization proposed that rectal neuroendocrine tumors (NETs) are classified as malignant tumors (4), and the 5-year survival rates for RNETs were 64.1% and 88% in Europe and North America, respectively (5–7).

RNETs were mostly limited to local (8) and endoscopic dissection for most cases, which was sufficient (9). However, lymph node (LN) metastases were found in nearly 10% of cases (10). Surgery with lymphadenectomy represents the gold standard for the curative treatment of localized disease with LN metastasis (11). Although tumor size, endoscopic aspect, CT appearance, etc. could predict LN metastasis (12), how to diagnose LN metastases accurately remains uncertain.

NETs typically express somatostatin receptors (SSTRs) on their cell membranes (13). Due to the high expression of SST in most NETs, SST imaging has become the current standard for staging and preoperative assessment of NET patients (14, 15). Of all methods available, PET-CT with 68Ga-labeled somatostatin analogs (SSAs) (68Ga‐DOTATATE, 68Ga‐DOTANOC, and 68Ga‐DOTATOC) showed the best mix of diagnostic accuracy (16). However, the predictive value of LN metastasis for SST imaging remains to be explored.

FDG is a glucose analog that is actively transported into the cell and subsequently remains in the cell. Tumor cells, due to their higher metabolic activity, usually have a higher FDG uptake than normal tissues (17). 18F-FDG PET/CT was considered the preferred radiotracer for G3 tumors, as well as for some high-grade G2 tumors (18). 18F-FDG PET-CT had a high diagnostic accuracy to identify progression in enteropancreatic NETs (19) and was a useful tool to predict the therapeutic effect in patients who underwent peptide receptor radionuclide therapy (20). However, 18F-FDG PET-CT shows high false negative findings, which could be related to the indolent tumor behavior of most NETs, such as G1 and low G2 tumors (21). The diagnostic role of 18F-FDG PET-CT is still controversial due to these conflicting results (22).

68Ga‐DOTANOC PET-CT seems to be superior to 18F-FDG PET-CT in the diagnostic performance of primary and LN metastases of pancreatic NETs (23, 24). However, whether 68Ga‐DOTANOC PET-CT can identify primary tumors and LN metastasis better than 18F-FDG PET-CT in patients with RNETs remains unclear.

In our study, we explored the diagnostic ability of 68Ga-DOTANOC PET-CT and 18F-FDG PET-CT for RNET primary tumors and regional LN group metastasis.

## Materials and Methods

### Patients

Patients who were diagnosed with RNETs from January 2014 to January 2021 were enrolled. The study was approved by the Ethics Committee of the First Affiliated Hospital of Sun Yat-sen University in China. All research was undertaken following the provisions of the Declaration of Helsinki. Patients with the following criteria were included: (1) confirmed RNETs according to 2019 World Health Organization (WHO) digestive system tumor classification criteria; (2) underwent 68Ga-DOTANOC PET-CT and 18F-FDG PET-CT within a 1-month period; and (3) absence of therapeutic intervention or change in disease status between the two PET studies. The exclusion criteria were as follows: (1) other colorectal malignancies; (2) uncertain diagnosis lacking pathology; and (3) long-acting radiolabeled somatostatin analog treatment in the 4 weeks prior to the study (25). All patients provided written informed consent and complied with the ethical guidelines in the Declaration of Helsinki.

### Pathological Diagnosis

The histological type of rectal NETs was defined according to the 2019 WHO classification (26), and tumor node metastasis (TNM) staging was characterized in our study according to the 2017 AJCC 8th edition (27). All NETs were graded according to the current guidelines of the 2019 WHO classification system based on mitotic counts and the Ki-67 labeling index. G1, G2, and G3 were classified according to the Ki-67 index. In brief, G1 was assigned to tumors with a mitotic rate <2/10 HPFs and/or a Ki-67 labeling index <3%, G2 to tumors with a mitotic rate 2 to 20/HPFs and/or a Ki-67 labeling index of 3% to 20%, and G3 to tumors with a mitotic rate >20/HPFs and/or a Ki-67 labeling index >20%.

### LN Group Classification

According to the Japanese Classification of Colorectal, Appendiceal, and Anal Carcinoma (28), regional LNs were mainly classified into pericolic, intermediate, main LN group, and lateral LN groups. For some cases, the surgeon selected the enlarged LN individually during surgery according to preoperative imaging.

### Reference Standard

Histopathology was taken as the reference standard.

### 68Ga-DOTANOC and 18F-FDG PET-CT Imaging

As Qiao He reported (29), no specific preparation of the patients was required before 68Ga-DOTANOC PET-CT and 18F-FDG examination. PET-CT imaging was performed with a Gemini GXL 16 PET scanner (Philips Healthcare). One hundred eleven to 185 MBq (3–5 mCi) 68Ga-DOTANOC or a dose of 5.18 MBq (0.14 mCi)/kg FDG was injected intravenously, and serial scanning was performed. Serial scanning from head to mid-thigh was performed approximately 45–60 min after the injection. Following low radiation dose CT acquisition with a slice thickness of 5 mm, PET acquisition was performed for 1.5 min per bed position for 7–8 beds using a slice thickness of 4 mm. CT-based attenuation correction of the emission data was employed. PET images were reconstructed by the Line of Response RAMLA algorithm.

18F-FDG and 68Ga-DOTANOC PET-CT studies were performed at least 24 h apart.

### Image Analysis

68Ga-DOTANOC and 18F-FDG PET-CT images were evaluated both visually and semi-quantitatively by two experienced nuclear medicine physicians in consensus. For the PET-CT studies, areas with focal activity greater than the background that could not be identified as physiological activity were considered to indicate tumor tissue (defined as visual assessment). The location and radioactivity uptake (maximum standard uptake value, SUVmax) of the lesions were observed or measured (defined as semiquantitative analysis).

### Statistical Analysis

Normally distributed variables were expressed as the means and standard deviations (SD), with variables not following a normal distribution presented as medians and interquartile ranges (IQRs) and categorical variables as frequencies and proportions. The Shapiro-Wilk test was used to test deviations from a normal distribution. The nonparametric analyses were performed using the Mann–Whitney U test. A receiver operating characteristic (ROC) curve was then constructed to determine the optimal SUVmax cutoff for predicting LN metastasis. A p value of <0.05 was considered statistically significant. The data analysis was performed using GraphPad Prism 8 software (GraphPad Software, Inc., La Jolla, CA, USA) and MedCalc statistical software.

## Results

### Patients Characteristics

**Figure 1:**
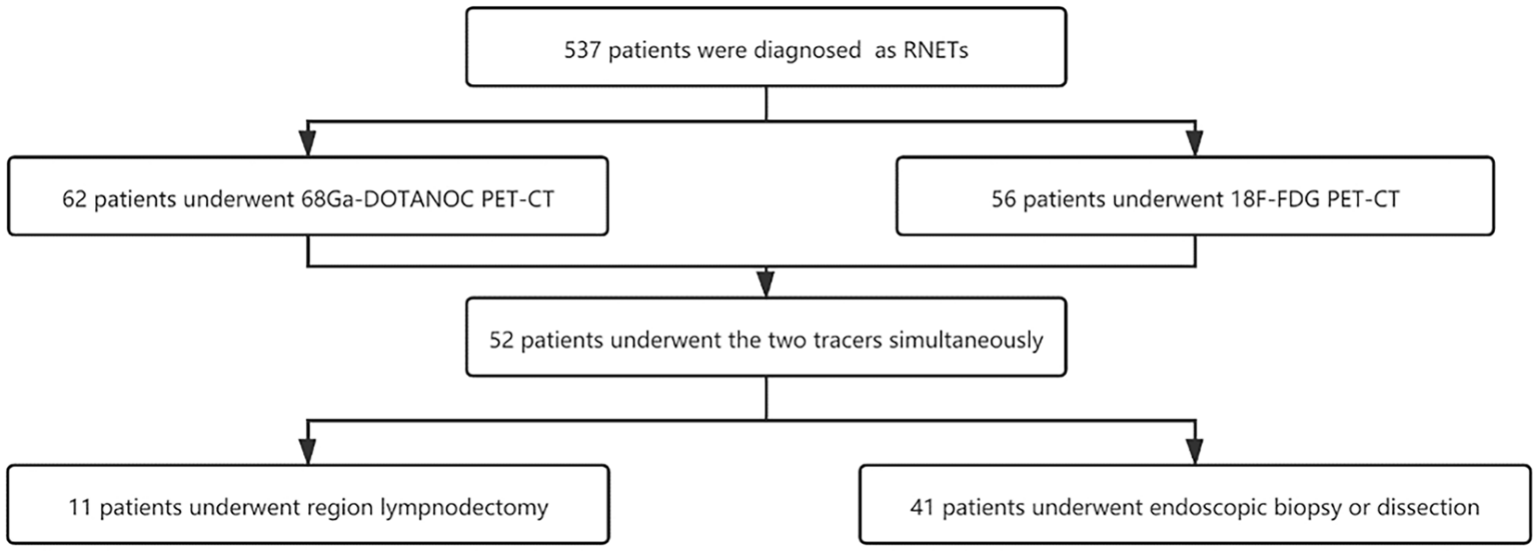
The flowchart of study patients.

A total of 52 patients who underwent both 68Ga-DOTANOC and 18F-FDG PET-CT scans were included in the study. Of the 11 patients who underwent regional LN dissection, 1 patient underwent salvage surgery after endoscopic submucosal dissection (ESD), 5 patients with distant metastases underwent surgery when intestinal obstruction occurred, and the other 5 patients underwent radical surgery. Another 41 patients underwent endoscopic biopsy or dissection only. More details are shown in **Figure 1** and **Table 1**.

**Table 1:** Basic clinicopathological characteristics in patients who underwent 68Ga-DOTANOC and 18F-FDG PET-CT.

Characteristics	value
Sex	
Male	32
Female	20
Age (years)	28-75
TNM stage	
I-III	11
IV	41
Grade	
G1	15
G2	31
G3	6

TNM, tumor node metastasis.

### Comparison of the Performance of 68Ga-DOTANOC and 18F-FDG PET-CT in Primary Tumors

#### Visual Assessment

Of the 52 patients included, 48 patients were pathologically diagnosed with NETs for the primary tumor, while the remaining 4 patients were determined to be negative by pathology for the primary tumor due to preoperative endoscopic dissection.

68Ga-DOTANOC PET-CT successfully identified 43/48 primary tumors with a sensitivity of 89.58% and 95.56% PPV, while 18F-FDG PET-CT identified 37/48 primary tumors with a sensitivity of 77.08% and 97.37% PPV. The sensitivity of 68Ga-DOTANOC PET-CT was not statistically different from that of FDG 18F-FDG PET-CT (p = 0.109). The combination of 68Ga-DOTANOC and 18F-FDG PET-CT could increase the sensitivity to 93.75%.

**Figure 2:**
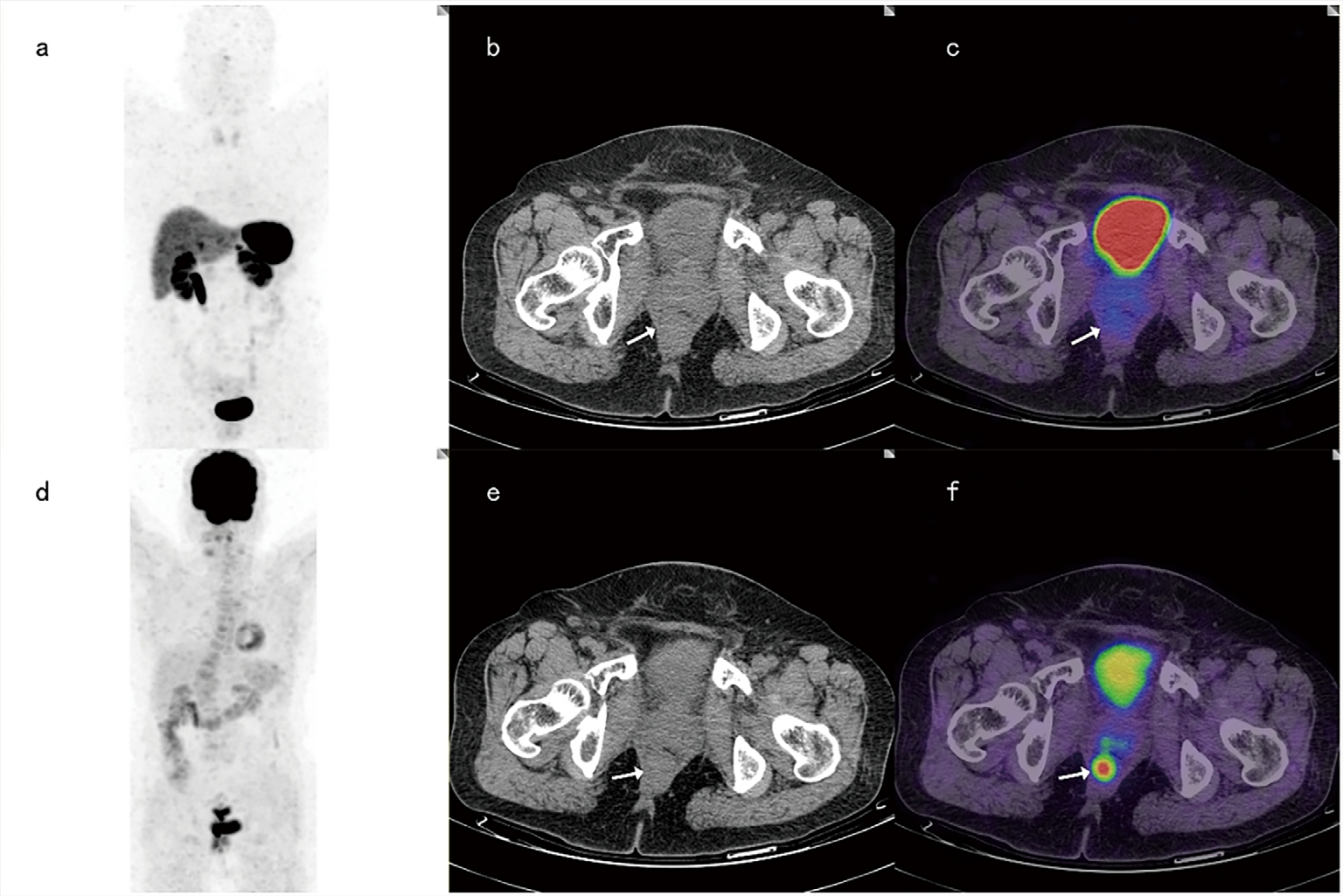
A 65-year-old female patient with rectal neuroendocrine carcinoma (Ki-67 was 90%). 68Ga-DOTANOC PET-CT images and corresponding MIP images **(A–C)** showed no focal uptake of 68Ga-DOTANOC, while 18F-FDG PET-CT imaging **(D–F)** showed focal uptake of 18F-FDG.

For 6 cases with G3 primary tumors, 68Ga-DOTANOC PET-CT identified 4 of these 6 patients and showed false negatives in 2 cases, while 18F-FDG PET-CT diagnosed 5 of these 6 patients and reported false negatives in one case (**Figure 2**). At the same time, among 42 patients with G1-2 primary tumors, 68Ga-DOTANOC PET-CT identified 39 patients (39/42), while 18F-FDG PET-CT identified only 32 patients (32/42) (p =0.039).

### The Value of 68Ga-DOTANOC and 18F-FDG PET-CT in Predicting LN Group Metastasis

Among the 11 patients who underwent regional lymphadenectomy, 10 patients were pathologically diagnosed with LN group metastasis. Forty-two groups of regional LNs were harvested after surgery, of which 18 groups were diagnosed with metastases. More details are shown in **Table 2**.

**Table 2:** General characteristics of patients who underwent region LN dissection.

Variables	Value
Sex	
Male	6
Female	5
Age (years)	38-70
TNM stage	
I-III	6
IV	5
Grade	
G1	2
G2	8
G3	1
LN group metastases	
Positive	18
Negative	24

TNM, tumor node metastasis; LN, Lymph node.

#### Visual Assessment

68Ga-DOTANOC PET-CT was true positive in 14 LN groups and true negative in 22 LN groups; thus, the sensitivity of 68Ga-DOTANOC PET-CT for detecting RNETs was 77.78%, and the specificity was 91.67%. Meanwhile, 18F-FDG PET-CT was true positive in 7 LN groups and true negative in 24 LN groups, with 38.89% sensitivity and 100% specificity. The overall sensitivity, specificity, and accuracy of 68Ga-DOTANOC and 18F-FDG PET-CT in predicting LN group metastasis are presented in **Table 3**.

**Table 3:** Sensitivity, specificity, positive and negative predictive value and accuracy for the prediction of LN group metastasis by 68Ga-DOTANOC and 18F-FDG PET through visual assessment.

	68Ga-DOTANOC PET	18F-FDG PET	p value
Sensitivity (%) (95% CI)	77.78 (52.36-93.59)	38.89 (17.30-64.25)	0.018
Specificity (%) (95% CI)	91.67 (73.00-98.97)	100	0.489
PPV (%) (95% CI)	87.5 (64.48-96.43)	100	1.000
NPV (%) (95% CI)	84.62 (69.68-92.94)	69.57 (55.20-80.92)	0.150
Accuracy (%) (95% CI)	85.71 (71.46-94.57)	68.57 (60.15-75.93)	0.175

PPV, positive predictive value; NPV, negative predictive value.

**Figure 3:**
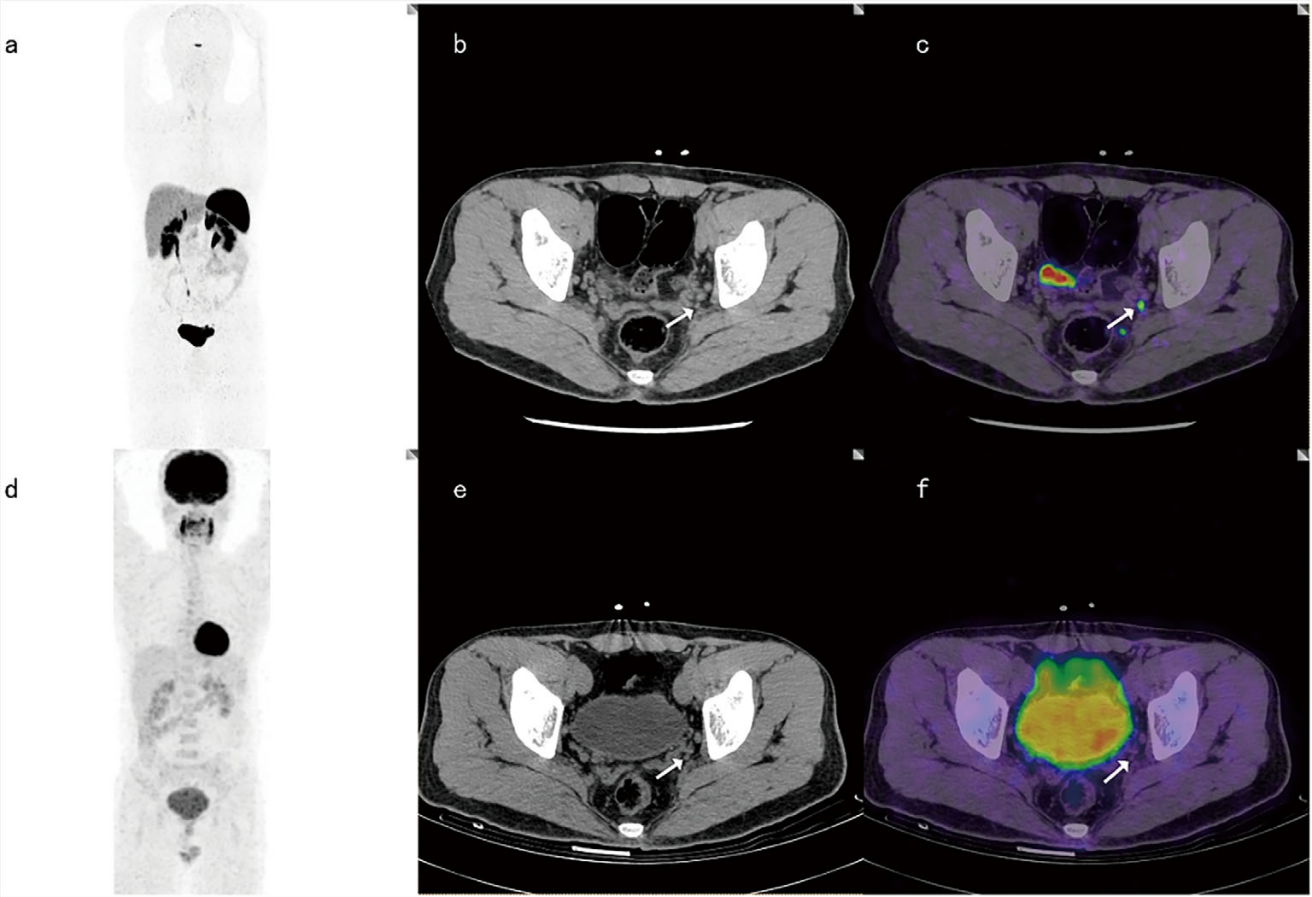
A 37-year-old male with LN metastasis adjacent to the left iliac blood vessel. The focal uptake of 68Ga-DOTANOC in LNs (as shown by the arrow) was obviously increased **(A–C)**, while the focal uptake of 18F-FDG was similar to that in the background **(D–F)**.

Among the 18 positive LN groups, both 68Ga-DOTANOC PET-CT and 18F-FDG PET-CT were true positive in 7 LN groups. Among the 24 negative LN groups, 18F-FDG PET-CT defined all true negatives (24/24), while 68Ga-DOTANOCPET-CT assessed 2 of 24 LN groups as false positives (2/24). Both 68Ga-DOTANOC and 18F-FDG PET-CT were positive in 7 LN groups and negative in 22 LN groups. However, discordance was noted in 13 groups between the two tracers (**Figure 3**).

#### Semiquantitative Assessment

**Figure 4:**
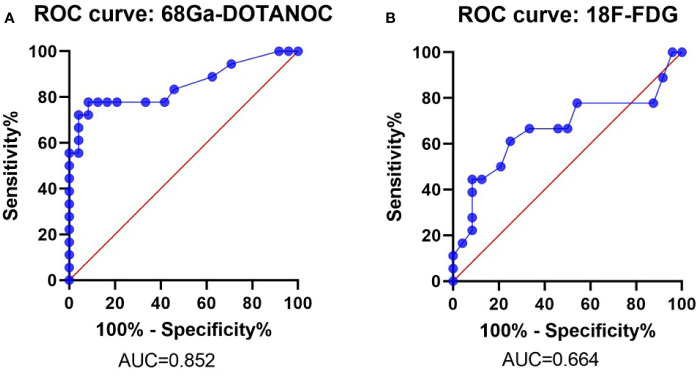
ROC Curve of the SUVmax of 68Ga-DOTANOC and 18F-FDG PET in predicting LN group metastasis. ROC Curve of 68Ga-DOTANOC PET, The AUC was 0.852 **(A)**; ROC Curve of 18F-FDG PET. The AUC was 0.664 **(B)**. ROC, Receiver Operating Characteristics; AUC, The area under the ROC curves.

ROC analysis showed that the optimal SUVmax cut-off value with the highest accuracy for predicting malignant nodes through 68Ga-DOTANOC PET was 2.25 with 77.78% sensitivity, 91.67% specificity, 87.50% PPV, 84.62% NPV and 85.71% accuracy. The AUC was 0.824 (95%CI:0.723-0.981) (**Figure 4A** and **Table 4**).

**Table 4:** Sensitivity, specificity, positive and negative predictive value, accuracy and AUC for the prediction of LN group metastasis by 68Ga-DOTANOC and 18F-FDG PET through semiquantitative assessment.

	68Ga-DOTANOC PET	18F-FDG PET	p value
Sensitivity (%) (95% CI)	77.78 (52.36-93.59)	61.11 (35.75-82.70)	0.278
Specificity (%) (95% CI)	91.67 (73.00-98.97)	75.00 (53.29-90.23)	0.121
PPV (%) (95% CI)	87.50 (64.48-96.43)	64.71 (45.54-80.08)	0.127
NPV (%) (95% CI)	84.62 (69.68-92.94)	72.00 (57.96-82.75)	0.274
Accuracy (%) (95% CI)	85.71 (71.46-94.57)	69.05 (52.91-82.38)	0.015
AUC (95% CI)	0.852 (0.723-0.981)	0.664 (0.485-0.844)	0.0583

PPV, positive predictive value; NPV, negative predictive value; AUC, Area under ROC curve.

Meanwhile, the optimal SUVmax cut-off value with the highest accuracy for predicting malignant nodes through 18F-FDG PET was 1.05 with 61.11% sensitivity, 75.00% specificity, 64.71% PPV,72.00% NPV and 69.05% accuracy. The AUC was 0.664 (95%CI: 0.485-0.844) (**Figure 4B** and **Table 4**).

## Discussion

This was the first study to evaluate the impact of 68Ga-DOTANOC and 18F-FDG PET-CT in predicting LN metastasis in patients with RNETs. Our study showed that 68Ga-DOTANOC PET-CT showed prospective ability to predict LN metastasis through visual assessment and semiquantitative assessment, which was better than 18F-FDG PET-CT. Meanwhile, the sensitivity of 68Ga-DOTANOC in primary tumors were better than those of 18F-FDG PET-CT.

In our study, the overall sensitivity of 68Ga-DOTANOC PET-CT was 89.58%, and the PPV was 95.56% in primary tumors. Even for G3 tumors, 4/6 of primary tumors could be detected by 68Ga-DOTANOC PET-CT. Several previous studies have shown that the sensitivity of 68GA-DOTANOC PET-CT for detecting gastrointestinal pancreatic primary lesions was 71.4%-94.4% (30–32), suggesting that 68Ga-DOTANOC had good diagnostic sensitivity. Meanwhile, 18F-FDG PET-CT identified 37/48 primary tumors with 77.08% sensitivity in our research, which was similar to the results of Zhang, P et al. (33). Other studies have shown that 18F-FDG PET-CT was not sensitive for diagnosing NETs (sensitivity 33%-66.7%) (19, 34, 35). In our study, the sensitivity of 68Ga-DOTANOC PET-CT was higher than that of 18F-FDG PET-CT for patients with G1-G2 tumors (p=0.039), indicating that 68Ga-DOTANOC was more reliable for the diagnosis of G1-G2 RNETs.

We also found that the combination of 68Ga-DOTANOC and 18F-FDG PET-CT slightly increased the sensitivity to 93.75% in detecting primary tumors. Similarly, a study by Partelli, S. et al. (22) also showed that 68Ga-DOTANOC PET-CT combined with 18F-FDG PET-CT could only slightly increase the sensitivity in pancreatic neuroendocrine tumors, suggesting that 18F-FDG PET-CT is unnecessary for detecting RNETs.

The current literature evaluating 68Ga-DOTANOC PET-CT for diagnosing LN metastasis is limited. Our research showed that 68Ga-DOTANOC PET-CT had 77.78% sensitivity and 91.67% specificity in both visual assessment and semiquantitative assessment for diagnosing LN group metastasis in RNETs, showing that the two evaluation methods were highly consistent. As reported by Ansquer, C (36). 68Ga-DOTANOC PET-CT had a sensitivity of 86.4% in midgut neuroendocrine tumors, and Niraj Naswa reported that 68Ga-DOTANOC had a sensitivity of 92.8% and specificity of 100% in diagnosing LN metastasis for gastroenteropancreatic neuroendocrine tumors (23), indicating that 68Ga-DOTANOC was a good tool for screening LN metastasis.

A study by Majala S. showed that only 33% of LN metastases could be diagnosed through 18F-FDG PET-CT in nonfunctional pancreatic neuroendocrine tumors (24). Meanwhile, another study showed that 68Ga-DOTANOC PET-CT had 94.2% sensitivity, 87.5% specificity, and 92.1% accuracy while 18F-FDG PET-CT had 25.7% sensitivity, 100% specificity, and 49% accuracy in gastroenteropancreatic neuroendocrine tumors (23). In concordance with the above research, our research showed that the sensitivity of 18F-FDG PET-CT for detecting LN group metastasis was only 38.89% in visual assessment and 61.11% in semiquantitative assessment with an AUC of 0.664, which was lower than the sensitivity and AUC of 68Ga-DOTANOC PET-CT (sensitivity was 77.78% and the AUC was 0.854). Therefore, we concluded that 68Ga-DOTANOC PET-CT was more suitable for screening LN group metastasis than 18F-FDG PET-CT.

68Ga-DOTANOC and 18F-FDG PET-CT had similar diagnostic capabilities in evaluating primary tumors but had different diagnostic capabilities in LN metastases. The result may be due to the active lymph node inflammation in the rectal network (37), which affects FDG uptake (38) and cause FDG PET is not reliable for detecting LN metastases of RNETs or maybe 68Ga-DOTANOC is actually better than FDG as N. Naswa reported (23).

Usually, in parallel with an increasing tumor proliferation rate (Ki-67 index), 68 Ga-DOTA- somatostatin receptor expression in NETs decreases (39). For NETs with Ki-67 greater than 15%, metabolic imaging with 18 FDG PET-CT is usually preferred rather than 68 Ga-DOTA-somatostatin analog PET-CT because of the low or absent somatostatin receptor expression in NET lesions (40). In our study, most patients had Ki-67 less than 15%, which may explain why the performance of 68Ga-DOTANOC PET-CT was better than 18F-FDG PET-CT. Therefore, 68Ga-DOTANOC PET-CT seems to be more suitable for RNET assessment than 18F-FDG PET-CT, especially in G1-G2 RNETs.

With the emergence of molecular imaging, surgeons are using molecular imaging to image-guided surgery (41). Hybrid detection modalities for image-guided surgery has been applied such as the application of indocyanine green (ICG)-99mTc-nanolloid for cancers (42). Similarly, Håkan Orlefors reported that 11C-5-Hydroxytryptophane PET can localize the NETs (43). However, the hybrid detection modalities for image-guided surgery in NETs are rare. SSTRs such as 68Ga-DOTANOC have high specificity, but whether it is suitable for guided surgery needs further study; meanwhile, combination of image-guided surgery and robot-assisted with laparoscopic surgery may reduce surgical complications in the future (44).

The strengths of the present study are that we evaluated simultaneously the primary tumors and regional LN group histologically of RNETs. However, there are some limitations. Firstly, the major limitation of the present study was the number of patients, as RNETs are rare; therefore, the conclusion should be treated carefully, and more cases need to be studied in the future. Secondly, as the study was retrospective, RCT research is needed.

## Data Availability Statement

The original contributions presented in the study are included in the article/supplementary material. Further inquiries can be directed to the corresponding author.

## Ethics Statement

The studies involving human participants were reviewed and approved by Ethics Committee of the First Affiliated Hospital of Sun Yat-sen University. The patients/participants provided their written informed consent to participate in this study.

## Author Contributions

ZW and GL designed this study. ZZ interpreted the patient data and drafted the manuscript. BZ and YW contributed to data collection. GL and ZXW revised the manuscript. All authors contributed to the article and approved the submitted version.

## Funding

This study was funded by the Natural Science Foundation of Guangdong Province, China (Grant Nos. 2016A030310155, 2017A030313577, and 2018A030313978) and the National Natural Science Foundation of China (Grant Nos. 81602049 and 81802342).

## Conflict of Interest

The authors declare that the research was conducted in the absence of any commercial or financial relationships that could be construed as a potential conflict of interest.

## Publisher’s Note

All claims expressed in this article are solely those of the authors and do not necessarily represent those of their affiliated organizations, or those of the publisher, the editors and the reviewers. Any product that may be evaluated in this article, or claim that may be made by its manufacturer, is not guaranteed or endorsed by the publisher.

## Abbreviations

NETs, neuroendocrine tumors; NENs, neuroendocrine neoplasms; RNETs, Rectal NETs; LN, lymph node; SSAs, somatostatin analogs; TNM, tumor node metastasis; SUVmax, maximum standard uptake value; IQRs, interquartile ranges; ROC, Receiver Operating Characteristics; CI, confidence interval; ESD, endoscopic submucosal dissection; PPV, positive predictive value; NPV, negative predictive value; AUC, The area under the ROC curves.
